# Strengthening Support Systems: Family Resources and Intergenerational Care for Grandmother Caregivers

**DOI:** 10.1093/geroni/igaf122.1171

**Published:** 2025-12-31

**Authors:** Gaynell Simpson

**Affiliations:** Georgia Gwinnett College, Lawrenceville, Georgia, United States

## Abstract

This study examines the perceptions of family resources among seven urban African American grandmother caregivers. Using an ethnographic approach, data were gathered through participant observation, field notes, interviews, and genograms to identify available and unavailable sources of support. Findings indicate that grandmothers relied primarily on family members who had resources to share, with instrumental and socioemotional support received in areas such as household chores, financial assistance, health-related needs, respite care, and crisis intervention. Even grandmothers facing significant social challenges found family members to be reliable in times of crisis. The study underscores the importance of intergenerational programs that strengthen family systems in grandparent-headed households. While some extended family members provided essential support, others were unable to contribute due to barriers such as poverty, medical conditions, or substance use. Minimal attention has been given to extended kin or non-kin who may serve as potential sources of support. Given the cultural tradition of African American families relying on kin networks, it is critical to assess the presence, roles, and functions of support structures within these families. Beyond individual and family-level interventions, macro-level advocacy is essential to address the broader social and economic inequities that contribute to caregiving challenges. Poverty, discrimination based on race, gender, age, and socioeconomic class, and structural inequalities shape the lived experiences of these caregivers. Social workers must engage in direct practice, policy development, and advocacy efforts to address the systemic barriers affecting African American grandparent caregivers and their families.

